# Marked Subchondral Bandlike Osteopenia on Radiography after Trauma and Inactivity: A Report of four Cases

**DOI:** 10.1155/2013/234278

**Published:** 2013-04-09

**Authors:** J. Gossner, B. W. Raab, J. Larsen, S. Breitkreuz

**Affiliations:** ^1^Department of Clinical Radiology, Evangelisches Krankenhaus Göttingen-Weende, An der Lutter 24, 37074 Göttingen, Germany; ^2^Radiologische & Nuklearmedizinische Gemeinschaftspraxis “Am Rosdorfer Weg 70a” Göttingen, 37071 Göttingen, Germany; ^3^Department of Trauma Surgery, Evangelisches Krankenhaus Göttingen-Weende, An der Lutter 24, 37074 Göttingen, Germany

## Abstract

We report about four cases of marked subchondral osteopenia on followup radiography after trauma and prolonged disuse. This localized form of disuse osteopenia has not been reported in details beside the followup imaging of talar neck fractures, where it is known as the “Hawkins sign.” Due to its unique morphology, it can be easily recognized as a benign finding in posttraumatic followup imaging and can be morphologically distinguished from severe complications like complex regional pain syndrome type 1 (Sudeck's disease) or periarticular osteopenia in infectious arthritis. It is important for the radiologist and orthopaedic surgeon to be aware of this form of disuse osteopenia in the proper clinical context.

## 1. Introduction

Subchondral bandlike osteopenia of the talus has been described after talus fracture and is known as the “Hawkins sign” [1]. It is a form of localized disuse osteopenia and develops about 6–8 weeks after trauma if vascular supply is intact [2]. Beside the followup after talus fracture this form of reactive osteopenia has received little attention. We observed marked subchondral osteopenia in the followup after trauma in 4 patients and would like to highlight this finding and to discuss pathogenesis and differential diagnosis to other diseases with focal osteopenia, especially complex regional pain syndrom (CRPS) type 1, partial transient osteoporosis or infectious arthritis.

## 2. Case Presentations

**Figure 1:**
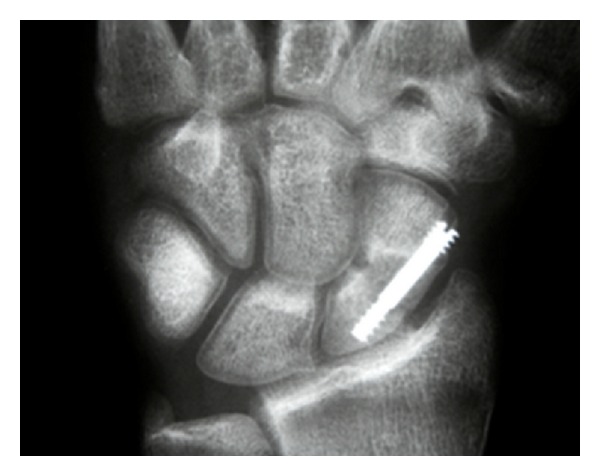
AP-radiograph of a 16-year-old patient in the follow up after screw fixation of a scaphoid fracture showing bandlike subchondral osteoporosis in all carpal bones.

*Case  1*. In a 16-year-old patient, a scaphoid fracture was treated with internal screw fixation. After a short time of splinting, the patient was adviced to reconvene usage. On followup radiography about 12 weeks after operation of the wrist, subchondral bandlike lucencies could be seen on all carpal bones. The cortex of the bones was intact (Figure 1). He reported reduced activity with the operated wrist. Clinical course was unremarkable, especially that there were no clinical or laboratory signs of infection or CPRS type 1.

**Figure 2:**
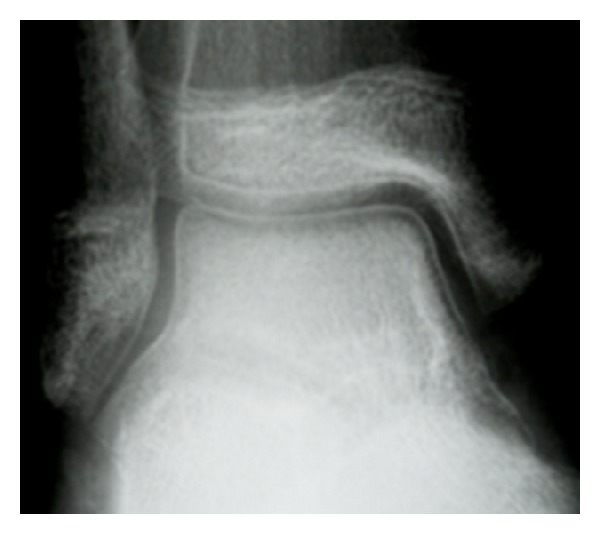
AP-radiograph of the ankle in a 12-year-old girl after immobilization after ankle sprain. Perfect subchondral bandlike osteoporosis of the talar dome can be seen.

**Figure 3:**
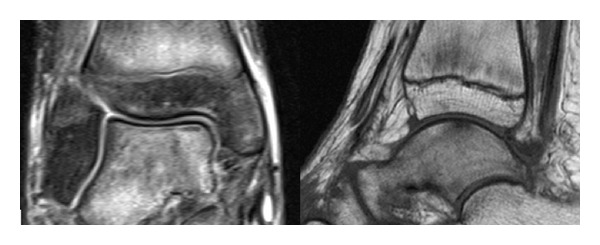
A coronal fat suppressed T2-weighted image and a sagittal T1-weighted image of a 12-year-old girl after ankle sprain showing diffuse bone marrow edema of the talus and the tibia. On conventional imaging, only subchondral bandlike osteoporosis of the talar dome could be seen (Figure 2).

*Case  2*. A 12-year-old patient presented at our emergency department with complaints of chronic instability of the ankle following a sprain. Because of the pain, she had reduced her normal activities. On the first radiograph no pathology could be seen. As in our first case, the clinical findings were unremarkable and especially, that no signs of inflammation were notable. On the followup radiographs, about 8 months after the first presentation, a marked bandlike subchondral osteopenia of the talus could be seen (Figure 2). An MRI scan of the ankle was performed subsequently. The cortex of the talar dome was intact in all sequences, and no articular damage was present. But a diffuse bone marrow edema of the talus but also of the distal metaphysis of tibia, especially in the fat suppressed T2-weighted images, was evident. There was no fracture line on T1-weighted images (Figure 3).

**Figure 4:**
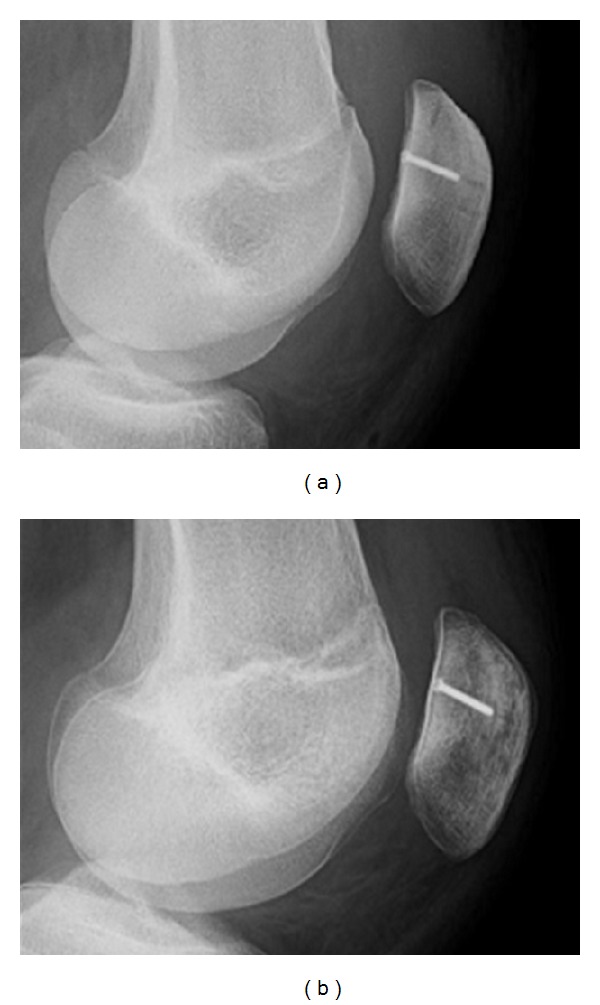
Serial axial radiographs of the patella after screw refixation of an osteochondral flake fracture in a 17-year-old patient. Followup imaging after operation (b) shows osteopenia with a subchondral predelication, this is in clear contrast to the immediately postoperative radiograph (a).

*Case  3*. A 17-year-old young man presented at our hospital after luxation of the patella. Computed tomography imaging showed an osteochondral flake of the articular surface of the patella. This fracture was treated with open reposition and screw fixation. The postoperative course was unremarkable. On followup imaging 6 weeks after the operation demineralization of the patella with a marked subchondral bandlike osteopenia could be found (Figure 4). There were no also no clinical signs of inflammation.

**Figure 5:**
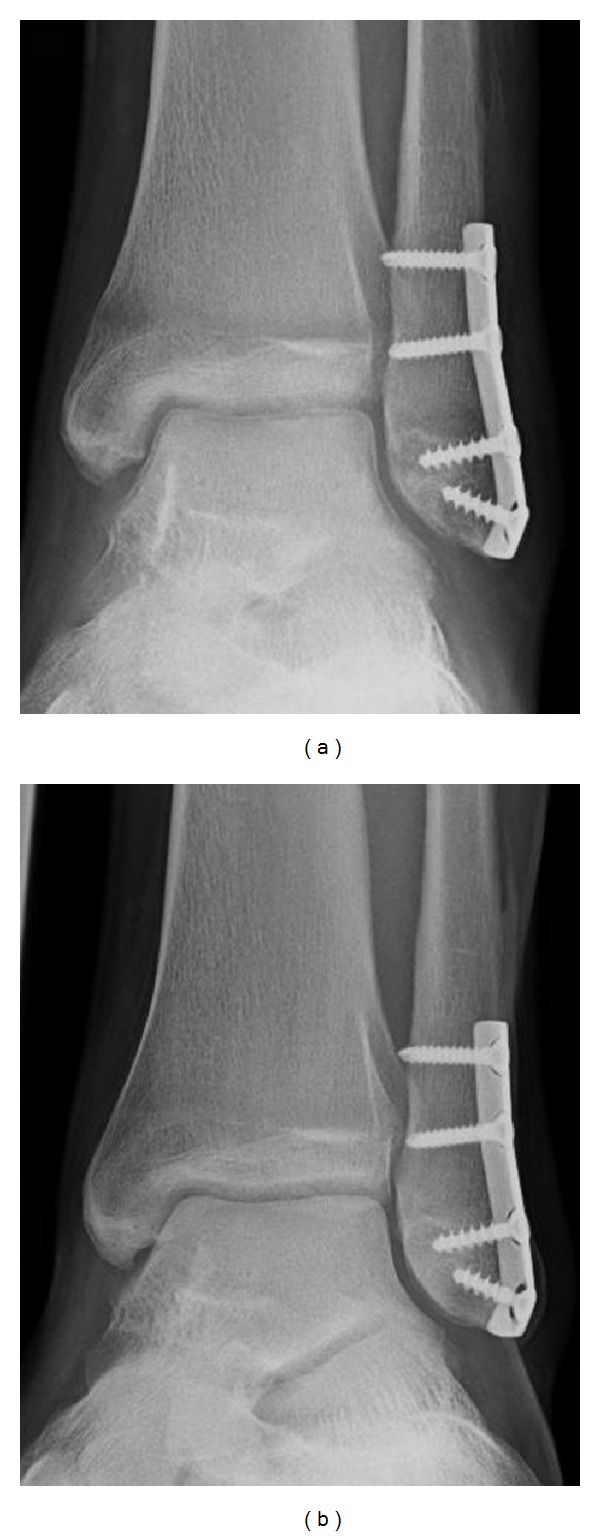
Serial radiographs of the left ankle after osteosynthesis of a distal fibula fracture. With immobility subchondral osteopenia of the talus as well as the distal tibial, epiphysis could be found (a) which disappeared after the resumption of normal activity (b).

*Case  4*. A 17 year old young man with a fracture of the distal fibula was treated with osteosynthesis. With immobility subchondral osteopenia of the talus as well as the distal tibial epiphysis could be found. The clinical course was unremarkable. On follow up 8 months later after resumption of normal activity the subchondral osteopenia disappeared (Figure 5). 

## 3. Discussion

Focal osteopenia can occur in a variety of diseases like infectious or rheumatoid arthritis and in a group of poorly understood disorders which include reflex sympathetic dystrophia, partial transient osteoporosis, transient osteoporosis of the hip, and regional migratory osteoporosis [3]. After trauma, an important differential diagnosis is CPRS type 1, also known as reflex sympathetic dystrophia or Sudeck's disease. The typical clinical picture includes continuing pain, hyperalgesia, edema, and changes in sudomotor activity. For diagnosis of CRPS type 1, the presence of a trauma or prolonged immobilization is requested [4]. Imaging shows diffuse periarticular osteopenia [5]. Another cause of focal osteopenia is partial transient osteoporosis and can be found in different regions of the body without the history of trauma or immobilization [6, 7]. Patients usually report about progressive and localized pain. The “classical” disease of this group of disorders is transient osteoporosis of the hip in pregnant women, which is characterized by its benign and self-limiting course. On conventional radiographs, diffuse osteoporosis of the femoral head can be seen [8]. Regional migratory osteoporosis is now considered a special form of partial transient osteoporosis. Another cause of periarticular osteopenia is infectious or rheumatoid arthritis [9]. In all these entities described above, bone marrow edema on MRI and an increased tracer uptake on skeletal scintigraphy can be found [10]. Bone marrow edema is an unspecific finding caused by capillary leakage, this can occur either as a result of impaired vascular drainage like in trauma and malignancy or by the presence of an increased bone marrow blood flow like in inflammation [11]. In a pathological study of biopsies from patients with transient osteoporosis of the hip, signs of a vasomotor response accompanying high bone turnover could be found [11]. So, it seems that a similarity in the pathophysiology of these above mentioned diseases with localized osteopenia is hyperemia. 

Localized osteopenia can also be found with inactivity, for example, after trauma. With reduced mechanical stress, there is increased bone turnover and resorption leading to osteopenia [12]. Hawkins observed in a large case series of talar neck fractures that bandlike osteoporosis of the talar dome can be found about 6–8 weeks after trauma in patients with intact vascular supply. In these cases, localized subchondral disuse osteopenia occurs; in contrast, in cases with avascular necrosis, progressive sclerotic areas can be found [2]. Bandlike subchondral osteopenia of the talus was, therefore, named Hawkins sign and conceptualized as a focal form of disuse osteopenia [1]. This bandlike subchondral osteopenia can also be observed only in parts of the talar dome if there is incomplete avascular necrosis [13]. The subchondral bone shows marked porosity with a high number of terminal arteries forming an extensive capillary and venous network which is important for cartilage nutrition. This is reflected in the fact that blood flow in the subchondral bone is up 10 times higher than in normal cancellous bone [14]. This may explain the finding of prominent subchondral bandlike osteopenia, because the accelerated bone turnover is likely to manifest first in regions with higher vascularity. The finding of bone marrow edema on MRI in our second case may add further evidence to the concept of high bone turnover, as it may reflect hyperemic bone marrow. 

The unique finding of bandlike subchondral osteopenia can be easily distinguished from the above mentioned diseases (CPRS type 1, transient osteoporosis, and septic arthritis), which are showing a more diffuse periarticular osteopenia. 

## 4. Conclusion

Bandlike subchondral osteopenia on follow up after trauma can not only be seen at the talar dome after talar neck fractures but also in other clinical scenarios and other parts of the skeleton. Due to its unique morphology, it can be easily recognized as a benign finding in posttraumatic followup imaging. It is important for the orthopaedic surgeon and radiologist to be aware of this form of disuse osteopenia in the proper clinical context and its advantageous indication of preserved blood flow.
